# A central role for ubiquitination within a circadian clock protein modification code

**DOI:** 10.3389/fnmol.2014.00069

**Published:** 2014-08-07

**Authors:** Katarina Stojkovic, Simon S. Wing, Nicolas Cermakian

**Affiliations:** ^1^Douglas Mental Health University Institute, McGill University, Montréal, QCCanada; ^2^Polypeptide Laboratory, Department of Medicine–McGill University Health Centre Research Institute, McGill University, Montréal, QCCanada

**Keywords:** circadian clock, clock gene, ubiquitin, ubiquitin ligase, deubiquitinase, stability

## Abstract

Circadian rhythms, endogenous cycles of about 24 h in physiology, are generated by a master clock located in the suprachiasmatic nucleus of the hypothalamus and other clocks located in the brain and peripheral tissues. Circadian disruption is known to increase the incidence of various illnesses, such as mental disorders, metabolic syndrome, and cancer. At the molecular level, periodicity is established by a set of clock genes via autoregulatory translation–transcription feedback loops. This clock mechanism is regulated by post-translational modifications such as phosphorylation and ubiquitination, which set the pace of the clock. Ubiquitination in particular has been found to regulate the stability of core clock components but also other clock protein functions. Mutation of genes encoding ubiquitin ligases can cause either elongation or shortening of the endogenous circadian period. Recent research has also started to uncover roles for deubiquitination in the molecular clockwork. Here, we review the role of the ubiquitin pathway in regulating the circadian clock and we propose that ubiquitination is a key element in a clock protein modification code that orchestrates clock mechanisms and circadian behavior over the daily cycle.

## INTRODUCTION: THE MOLECULAR CIRCADIAN CLOCK

Circadian rhythms are endogenous ∼24 h cycles in physiology and behavior generated by a master clock in the suprachiasmatic nucleus of the hypothalamus, and clocks located in most other tissues. Circadian clocks enable organisms to anticipate predictable daily occurrences, such as changes in light, temperature, or food availability (Dibner et al., 2010). The importance of circadian clocks is illustrated by the impacts of circadian disruption in humans. For example, shift work increases the risk of developing various illnesses, such as mental disorders, metabolic syndrome, and cancer (Evans and Davidson, 2013).

**FIGURE 1:**
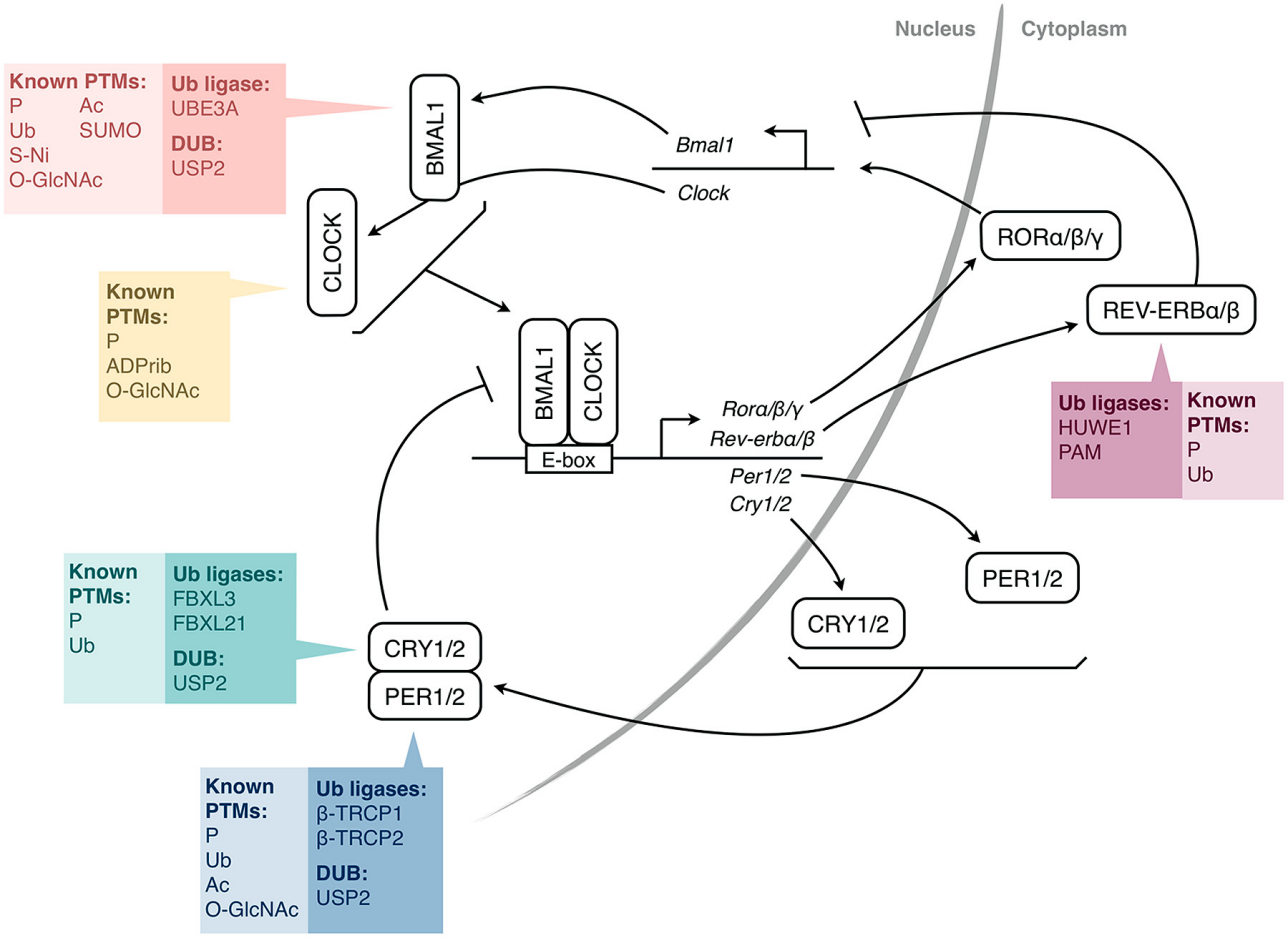
**Simplified molecular mechanism of the mammalian circadian clock.** The transcription factors CLOCK and BMAL1 activate the expression of *Per* and *Cry* genes through E-box elements in their promoters. PER and CRY proteins form complexes and feedback negatively on CLOCK/BMAL1 activity, and thus, on their own expression. CLOCK/BMAL1 also activate genes encoding nuclear receptors of the REV-ERB and ROR families, which regulate the expression of *Bmal1* (and also *Cry1* and *Clock*). For each protein or pair of proteins, the colored boxes list the post-translational modifications (PTMs) that have been identified so far, as well as the ubiquitin-modifying enzymes shown to be involved (HUWE1, PAM, β-TRCP1/2 are also called ARF-BP1, MYCBP2, FBW1A/B, respectively). For simplicity, a single box per protein is shown, irrespective of the subcellular localization of the PTMs. P, phosphorylation; Ub, ubiquitination; Ac, acetylation; SUMO, SUMOylation; O-GlcNAc, addition of β-D-*N*-acetylglucosamine; S-Ni, S-nitrosylation; ADPrib, ADP-ribosylation; PER, Period; CRY, Cryptochrome; ROR, Retinoic acid receptor-related orphan receptor; DUB, deubiquitinating enzyme.

At the molecular level, the circadian clock relies on self-sustained transcription-translation feedback loops involving “clock genes” (**Figure 1**; Duguay and Cermakian, 2009). In mammals, CLOCK and BMAL1 dimerize and activate *Period (Per) 1* and *2* and *Cryptochrome* (*Cry*) *1* and *2* genes. The PER1/2 and CRY1/2 proteins then enter the nucleus and inhibit the activity of CLOCK/BMAL1, thereby repressing their own transcription. However, the mechanism is more complex, with additional interlocking feedback loops, including one that involves the induction of the *Rev-erb* and *Ror* genes, whose protein products regulate *Bmal1* gene transcription. One consequence of these feedback loops is that the mRNAs and proteins of many clock genes present circadian rhythms in their abundance. Moreover, hundreds of clock-controlled genes, which do not participate in the clock mechanism, but whose transcription is under the control of the clock molecular machinery, also present rhythms at the RNA and protein levels (Storch et al., 2002; Yan et al., 2008), thus linking the circadian clock with cellular physiology.

The timing of these feedback loops is dictated by post-translational modifications (PTMs; Gallego and Virshup, 2007; Duguay and Cermakian, 2009). Indeed, clock proteins are subject to phosphorylation, ubiquitination, acetylation, SUMOylation, and other PTMs (**Figure 1**). Ubiquitination is of particular interest due to the diversity of signals that it can generate. In particular, its direct role in determining protein half-life is crucial for proteins with a daily rhythm in abundance. In this article, we review the current state of knowledge on ubiquitination of clock proteins and their ubiquitin-modifying enzymes in animal models, with a special focus on the mammalian clock (**Table 1**).

**Table 1:** Ubiquitin-modifying enzymes involved in the regulation of mammalian clock proteins.

Clock protein	Mammalian enzyme	Phenotype of mice, tissues, or cells upon loss-of-function mutation or knock-down of ubiquitin-modifying enzyme	*Drosophila* homolog	References
*Ubiquitin ligases*			
CRY1/2	FBXL3	Long free-running period of locomotor activity rhythms, cultured fibroblasts and SCN; stabilized CRYs; dampened and delayed rhythms of *Per* and *Cry* mRNAs; phenotype partly rescued by *Fbxl21* loss-of-function.		Godinho et al. (2007), Siepka et al. (2007), Hirano et al. (2013), Yoo et al. (2013)
	FBXL21	Normal or short free-running period of locomotor activity rhythms; short period of cultured fibroblasts, SCN and pituitary; destabilized CRYs (when subcellular fractions were studied, the mutation stabilized CRY1 in cytoplasm and destabilized it in the nucleus); mutation partly rescues *Fbxl3* loss-of-function.		Dardente et al. (2008), Hirano et al. (2013), Yoo et al. (2013)
PER1/2	β-TRCP1 (FBW1A), β-TRCP2 (FBW1B)	Dampened or long-period rhythms in fibroblasts; stabilized PERs; *β-Trcp1* KO mice have no circadian-related phenotype.	SLIMB	Eide et al. (2005), Shirogane et al. (2005), Reischl et al. (2007), Ohsaki et al. (2008)
REV-ERBα	HUWE1 (ARF-BP1)	Stabilized REV-ERBα, decreased *Bmal1/Cry1* expression (knock-down of both HUWE1 and PAM together).	CG8184	Yin et al. (2010)
	PAM (MYCBP2)	Stabilized REV-ERBα, decreased *Bmal1/Cry1* expression (knock-down of both HUWE1 and PAM together).	Highwire	Yin et al. (2010)
	FBXL3?	Long-period phenotype of *Fbxl3* mutants rescued by *Rev-erbα* KO; dampened but more sustained REV-ERBα levels in *Fbxl3* mutants, and prolonged REV-ERBα transcriptional activity.		Shi et al. (2013)
BMAL1	UBE3A	Dampening and longer period of circadian rhythms in cultured fibroblasts.	dUBE3A	Gossan et al. (2014)
*Deubiquitinating enzymes*			
CRY1	USP2	Decreased CRY1 protein levels in liver (with *Usp2* knock-down).		Tong et al. (2012)
PER1	USP2	Slightly elongated free-running period of locomotor activity rhythms; altered response to light; altered clock gene expression and increased levels of ubiquitinated PER1 in fibroblasts; no change in PER1 stability; alteration in the timing of PER1 intracellular localization.		Yang et al. (2012, 2014)
BMAL1	USP2	Normal free-running period and slightly altered light response; reduced BMAL1 levels in the SCN.		Lee et al. (2008), Scoma et al. (2011)

## UBIQUITINATION IN THE CIRCADIAN CLOCK

### UBIQUITINATION OF CRYPTOCHROMES BY FBXL UBIQUITIN LIGASES

*N*-Ethyl-*N*-nitrosourea screens led to the discovery of mice exhibiting free-running periods of locomotor activity rhythms ∼2–3 h longer than normal (Godinho et al., 2007; Siepka et al., 2007). These mice had loss-of-function mutations in the gene encoding the F-box protein FBXL3. Loss of FBXL3 activity leads to CRY protein stabilization due to decreased ubiquitination (Siepka et al., 2007). Further work on FBXL3 revealed that ubiquitination of CRY1/2 by the FBXL3-containing SCF E3 ubiquitin ligase complex was necessary for the timely degradation of the CRY proteins and the reactivation of BMAL1/CLOCK (Busino et al., 2007). A prolonged inhibition of BMAL1/CLOCK-mediated transcription in the mutant mice leads to reduced peak levels and delayed rhythms of the *Per* and* Cry* mRNAs in mutant mouse SCN, cerebellum, and liver (Godinho et al., 2007; Siepka et al., 2007).

Interestingly, FBXL3 cannot undergo SCF complex formation in the absence of its CRY substrates (Yumimoto et al., 2013). X-ray crystallography revealed that FBXL3 binds to the FAD-binding pocket of mammalian CRY, which may also be bound by FAD or PER proteins (Czarna et al., 2013; Xing et al., 2013), which suggests a mechanism for the protection of CRYs from degradation in the presence of PER (Yagita et al., 2002).

An FBXL3 paralog, FBXL21, was identified in sheep, where it was also found to bind to CRY1, thereby affecting transcriptional activation by CLOCK/BMAL1 (Dardente et al., 2008). Despite the high similarity between FBXL3 and FBXL21, they appear to have non-redundant roles within the clock. Indeed, while *Fbxl3* gene mutant or knock-out (KO) mice display a long free-running period of locomotor activity rhythms, *Fbxl21*-mutant or KO mice present either a short (Yoo et al., 2013) or a normal (Hirano et al., 2013) period. Moreover, when the mutant lines are crossed, the *Fbxl21* mutation attenuates the long-period phenotype of *Fbxl3*-mutant mice.

The distinct roles of the FBXL proteins may be based on the timing of their expression and that of their substrates. While *Fbxl3* is expressed at constant levels over the day, *Fbxl21* expression has a pronounced circadian rhythm in the mouse SCN, with a peak by the end of the subjective day (Dardente et al., 2008), thus restricting its action to only part of the cycle. Interestingly, while FBXL3 protein levels do not vary over time, its action on CRYs is conditional on their phosphorylation by AMPK, whose expression and nuclear abundance vary over the day (Lamia et al., 2009). Ligase intracellular localization also plays a role: while FBXL3 protein is restricted to the nucleus, FBXL21 is located both in the nucleus and cytoplasm (Hirano et al., 2013; Yoo et al., 2013). The work of both laboratories supports a two-step mode of action of FBXL21. First, FBXL21 allows CRYs to accumulate in the cytoplasm. This occurs when CRY levels rise around the end of the day or beginning of the night. Shortly thereafter, after CRYs have entered the nucleus, FBXL21 might counteract FBXL3: FBXL21 binds CRYs more stably and with a higher affinity than FBXL3, suggesting that FBXL21 may in part stabilize CRYs by preventing FBXL3 binding (Yoo et al., 2013). Then, when FBXL21 levels have decreased, FBXL3 can finally act on CRYs and target them to degradation. It is interesting to note that if this model is further confirmed, the roles of FBXL21 in the clock will turn out to be partly non-degradative (regulation of nuclear entry, protection from the action of another F-box protein, FBXL3), in contrast to other ubiquitin ligases involved in the clock, which target clock proteins to proteasomal degradation. Finally, CRY ubiquitination mechanisms might be even more complex, as it was suggested that another ubiquitin ligase might be involved in regulating CRY accumulation (Kurabayashi et al., 2010; Hirano et al., 2013).

### UBIQUITINATION OF PERIOD PROTEINS BY β-TRCP UBIQUITIN LIGASES

In *Drosophila*, the F-box component of an SCF ligase, SLIMB, was shown to be critical for ubiquitination and degradation of PER protein over the course of the circadian cycle (Grima et al., 2002; Ko et al., 2002). SLIMB binds to PER after its phosphorylation by Doubletime (DBT), in particular on serine 47 within the SLIMB recognition site (Chiu et al., 2008).

SLIMB has two homologs in mammals, β-TRCP1/FBW1A and β-TRCP2/FBW1B. Similarly to the action of DBT and SLIMB on PER in the fly, β-TRCP1/2 are recruited to PER2 following phosphorylation of this protein by the kinases CK1δ and CK1ε (mammalian homologs of DBT), which leads to polyubiquitination and subsequent degradation of PER2 (Eide et al., 2005). Indeed, expression of a dominant negative form of β-TRCP leads to the inhibition of PER2 ubiquitination and degradation. β-TRCP1/2 also interact with PER1 in a CK1ε-dependent manner and a knockdown of both β-TRCPs was found to stabilize PER1, and reduce levels of transcriptional activation by CLOCK/BMAL1 (Shirogane et al., 2005). Accordingly, preventing the action of β-TRCP1/2 on PER proteins leads to long-period or dampened circadian rhythms in cultured fibroblasts (Reischl et al., 2007; Ohsaki et al., 2008). Surprisingly though, mice lacking β-TRCP1 neither show alteration in circadian locomotor behavior nor differences in SCN PER2 levels when compared to WT controls, suggesting either that the SCN clock behaves differently from clocks in fibroblasts or that there is redundancy at the level of the ubiquitin ligases (Ohsaki et al., 2008). Finally, similarly to PER proteins protecting CRYs (see Ubiquitination of Cryptochromes by FBXL Ubiquitin Ligases), PER proteins are protected from ubiquitination and degradation upon association with CRYs (Yagita et al., 2002).

### UBIQUITINATION OF REV-ERBα

The stability of REV-ERBα is also regulated by a sequence of phosphorylation, ubiquitination, and proteasomal degradation. Indeed, REV-ERBα is stabilized following phosphorylation by GSK3β (Yin et al., 2006). Treating cells with lithium, a GSK3β inhibitor, leads to the quick degradation of REV-ERBα and therefore, to increased expression of *Bmal1* (**Figure 1**). Subsequent work identified HUWE1/ARF-BP1 and PAM/MYCBP2 as E3 ligases involved in this lithium-induced REV-ERBα degradation (Yin et al., 2010). Their depletion in cells stabilized REV-ERBα, decreased *Bmal1* gene expression, and disrupted oscillations of other clock genes. HUWE1 and PAM may not be the only ubiquitin ligases acting on REV-ERBα. In *Fbxl3*-mutant mice, REV-ERBα levels are higher and consequently its repression of *Bmal1* and *Cry1* genes is enhanced. Creation of double-mutant *Fbxl3*/*Rev-erbα*^-/-^ mice rescues the *Fbxl3*-mutant phenotype (Shi et al., 2013) indicating that FBXL3, in addition to its role on CLOCK/BMAL1-mediated transcription via destabilization of CRYs, also has an effect on REV-ERBα-mediated repression of target genes. Although this effect may be indirect, it does indicate a role for this F-box protein as a coordinator of different clock transcription factors.

### UBIQUITINATION OF BMAL1

Many studies have indicated a tight regulation of BMAL1 stability. Indeed, BMAL1 undergoes different phosphorylation events that either target it for ubiquitination and degradation (e.g., GSK3β, Sahar et al., 2010) or on the contrary for deubiquitination and stabilization (e.g., PKCγ, Zhang et al., 2012). Importantly, BMAL1 ubiquitination and proteasome-mediated proteolysis appear to coincide with the time of highest transcriptional activity (Kwon et al., 2006; Lee et al., 2008; Stratmann et al., 2012), whereas in conditions where CLOCK/BMAL1 activity is repressed (e.g., presence of CRYs), BMAL1 is stabilized (Kondratov et al., 2006; Dardente et al., 2007). However, no BMAL1-specific ubiquitin ligase had been uncovered until a recent report, which described UBE3A as an E3 ligase that binds and destabilizes BMAL1 (Gossan et al., 2014). Knockdown of this ligase in mammalian cells and in *Drosophila* clock neurons leads to a strong dampening of circadian oscillations or even arrhythmicity.

## SUMOylation IN THE CIRCADIAN CLOCK

The small ubiquitin-related modifier (SUMO) proteins also play a role in the clock. Like other PTMs, SUMOylation is reversible and the conjugation/deconjugation mechanisms are reminiscent of the ubiquitin pathway (Muller et al., 2001). In contrast to ubiquitination though, SUMOylation does not directly target proteins for degradation but rather regulates other functions such as nuclear localization, protein–protein interactions, transcriptional activity and, interestingly, ubiquitination itself (Desterro et al., 1998; Buschmann et al., 2000).

SUMOylation was first implicated in the clock following the discovery of a SUMOylation consensus motif in BMAL1 (Cardone et al., 2005). Co-expression of BMAL1 and SUMO showed that BMAL1 could indeed be SUMOylated. In the liver, this occurs in a rhythmic manner, with peak SUMOylation in the second half of the light phase. This timing coincides with peak BMAL1 phosphorylation and activity, suggesting an interplay between these PTMs. In further support of this, a functional CLOCK protein is required for both BMAL1 SUMOylation and phosphorylation (Kondratov et al., 2003; Cardone et al., 2005; Dardente et al., 2007). SUMOylated BMAL1 is most abundant when the CLOCK/BMAL1 targets *Dbp* and *Rev-erbα* show their highest mRNA levels, again supporting that SUMOylation of BMAL1 is involved in its transcriptional activity (Lee et al., 2008). Indeed, BMAL1 binding to the *Dbp* promoter was reduced when the lysine required for SUMOylation was mutated (Lee et al., 2008). Interestingly, SUMOylation of BMAL1 is a prerequisite for its subsequent ubiquitination, again highlighting the interplay of different PTMs in the circadian clock.

## DEUBIQUITINATION IN THE CIRCADIAN CLOCK

Given the importance of ubiquitination within the clock, it appears reasonable to assume that deubiquitination plays a role as well. Interestingly, the mRNA levels of a deubiquitinating enzyme (DUB), ubiquitin-specific protease 2 (USP2), show rhythmicity in most tissues examined (Kita et al., 2002; Storch et al., 2002; Yan et al., 2008). This is notable, because among the hundreds of clock-controlled transcripts, only a small minority cycles in multiple locations. The circadian rhythm of *Usp2* is blunted in *Clock* mutant and *Bmal1* KO mice (Oishi et al., 2003; Molusky et al., 2012b), and the *Usp2* promoter is activated by CLOCK/BMAL1 (Molusky et al., 2012b), indicating that *Usp2* is a direct target of these transcription factors. In addition to its circadian regulation, *Usp2* expression is also induced by starvation and it was therefore proposed that USP2 integrates nutritional and circadian timing cues (Molusky et al., 2012b). In turn, liver USP2 appears to be involved in the generation of a diurnal rhythm in glucose metabolism (Molusky et al., 2012a).

However, the circadian role of USP2 is not limited to mediating the rhythmic control of cellular processes by the molecular clock. Since the short list of genes rhythmic in multiple tissues is enriched for clock components, USP2 was hypothesized to exert a role within the clock mechanism. To address this, *Usp2* KO mice were generated by two laboratories. In one case, they revealed no alteration of the free-running period of locomotor rhythms (Scoma et al., 2011). In contrast, our *Usp2* KOs display a period longer than WT littermates (Yang et al., 2012), implying a role within the clockwork. In line with this, the absence of USP2 affects the mRNA levels of several clock genes (Scoma et al., 2011; Yang et al., 2012, 2014), and USP2 interacts with clock proteins. In our hands, whereas it forms a complex with several clock proteins, USP2 directly binds only to PER1 (Yang et al., 2012). Accordingly, PER1 is deubiquitinated in the presence of USP2, but notably, this does not lead to PER1 stabilization. Instead, USP2 appears to regulate PER1 intracellular localization (Yang et al., 2014). Interestingly, the only other DUB that to our knowledge has been implicated in clock mechanisms, *Drosophila* USP8, also seems to act in a non-degradative manner: it deubiquitinates CLOCK, thereby inhibiting transcriptional activity of CLOCK/CYCLE (CYCLE is the *Drosophila* homolog of BMAL1; Luo et al., 2012).

In contrast, the work of other groups showed a stabilization of other clock proteins due to deubiquitination by USP2. BMAL1 levels are lower in the SCN of *Usp2* KO mice (Scoma et al., 2011), whereas in cultured cells, USP2 stabilized BMAL1 (Scoma et al., 2011) and reduced its ubiquitination (Lee et al., 2008). Interestingly, a report suggested the involvement of PKCγ-triggered deubiquitination of BMAL1 in the resetting of peripheral clocks by feeding schedules, but the DUB involved in this pathway remains unknown (Zhang et al., 2012). In addition to PER1 and BMAL1, USP2 deubiquitinates CRY1 in cultured cells in response to a serum shock, and in the mouse liver, *Usp2* knockdown increases CRY ubiquitination and decreases CRY1 protein levels (Tong et al., 2012).

Data also support a role for USP2 in the response of the clock to external cues. We found that *Usp2* KO mice exhibit larger phase delays than WT mice after light treatment in the first part of the night, and reduced phase advances, upon light treatment later in the night (Yang et al., 2012). Thus, USP2 appears to be involved in the response of the SCN clock to light, which is also supported by data of Scoma et al. (2011), which show increased phase-shifting in response to low irradiance light in the early night. USP2 may also mediate the response of the clock to inflammation, as the expression of the gene is increased in response to TNFα treatment, and CRY1 protein induction in response to this cytokine is abrogated when *Usp2* expression is knocked down (Tong et al., 2012).

Together, these studies ascribe a pivotal role to USP2, and deubiquitination in general, not only in the circadian clock mechanism, but also as an integrator of environmental and physiological signals, and in output pathways linking the molecular clockwork to cellular and physiological functions.

## A CLOCK PROTEIN MODIFICATION CODE?

Overall, the work described above underscores the importance of PTMs within the circadian timing mechanism. Given that different modifications often converge on the same clock protein, we propose the existence of a *clock protein modification code* whereby the fate/function of a given protein is determined by the precise combination and/or the consecutive occurrence of different PTMs. This clock protein modification code is proposed to exist at different levels:

1. Interplay of different PTMs: PTMs often occur sequentially. In particular, there are numerous examples of phosphorylation at specific sites being a pre-requisite for subsequent ubiquitination of the target protein (Cardozo and Pagano, 2004), as occurs in many clock proteins. Another example of sequential modification is the SUMOylation of BMAL1 as a pre-requisite for its ubiquitination (Lee et al., 2008). Moreover, different combinations of PTMs on a protein can lead to distinct outcomes. For example, dual SUMOylation and ubiquitination of BMAL1 result in the BMAL1 localization to the nuclear bodies and active transcription (Lee et al., 2008), whereas later in the circadian cycle, additional events, possibly including further ubiquitination, lead to degradation of the protein. Combinations of different phosphorylation events can also regulate protein fate differentially: for example, in *Drosophila*, PER phosphorylation by DBT is modulated by prior action of another kinase, NEMO, and consequently, these kinases have opposing effects on PER stability (Chiu et al., 2011).
2. Ubiquitin code: There is a large diversity in the ubiquitination of proteins (Heride et al., 2014): they can be mono- or polyubiquitinated; in the latter case, ubiquitin chains can be linear or branched, and the linkages between ubiquitin monomers can be via different lysines. These different ubiquitination states can be generated by various ligases/conjugating enzymes and DUBs. This ubiquitin code can be read by proteins containing ubiquitin-binding domains (UBDs). The effects of ubiquitination can therefore be diverse depending on the type of modifications and the presence of particular UBD-containing proteins: not only targeting to the proteasome, but also regulation of intracellular localization, activity, protein–protein interaction, etc. (Komander et al., 2009). As non-degradative functions of clock protein ubiquitination have started to be identified (see previous sections), it is now important to characterize precisely the ubiquitin code (location and type of ubiquitination) on clock proteins and identify the specific UBD-containing proteins that recognize the code and translate it into specific effects on clock proteins.
3. PTMs around the clock: PTMs of clock proteins are orchestrated across the 24 h cycle. For example, BMAL1 undergoes a series of PTMs associated with a variation in activity and partner binding. Peak phosphorylation and SUMOylation of BMAL1 occurs in the late subjective day in mouse peripheral tissues (Cardone et al., 2005; Lee et al., 2008), and SUMOylation is a pre-requisite for ubiquitination (Lee et al., 2008). The occurrence of these PTMs coincides with peak transcriptional activity of the CLOCK/BMAL1 dimer (Ripperger and Schibler, 2006; Stratmann et al., 2012). Further, this appears to be regulated by another PTM, O-GlcNAcylation, which opposes the ubiquitination of BMAL1 (Li et al., 2013). This maximal activity of CLOCK/BMAL1 results in expression of CRY proteins that then repress CLOCK/BMAL1, at a time that is synchronous with the BMAL1 dephosphorylation and stabilization (Kwon et al., 2006; Dardente et al., 2007). BMAL1 acetylation by CLOCK also occurs at this time and leads to increased recruitment of CRY (Hirayama et al., 2007). CRYs themselves are good examples of substrates for sequential PTMs over the 24 h day and across the progression of the clock feedback loop (see Ubiquitination of Cryptochromes by FBXL Ubiquitin Ligases). Therefore, each clock protein undergoes a daily wave of PTMs, in a sequential and often conditional manner, which determines the expression, localization, and activity of the protein and its partners.

## CONCLUSION

In conclusion, ubiquitination and deubiquitination are involved in the regulation of key core clock components. On one hand, ubiquitin ligases are selectively acting on one or a few clock proteins. A given clock protein can even be the target of two or three different E3 ligases, depending on the time of day and cellular compartment. On the other hand, DUBs seem less specific, and only one was identified as a mammalian clock component so far: USP2. This DUB regulates the stability and function of PER1, CRY1, BMAL1 and perhaps other clock proteins, as well as components of the input and output pathways of the clock. Moreover, there is a complex interplay of ubiquitination with other PTMs. It will be crucial in future years to precisely define ubiquitin chain configurations and conjugation sites on clock proteins, to unravel the precise regulation of their addition and removal and identify all the actors involved. Furthermore, ubiquitination becomes an attractive drug target. Indeed, recent chemical screens of compounds binding CRY proteins have identified molecules modulating their ubiquitin-induced degradation (Hirota et al., 2012), suggesting the possibility of therapeutic resetting of the circadian clock by drug-mediated ubiquitin modulation of clock components.

## Conflict of Interest Statement

The authors declare that the research was conducted in the absence of any commercial or financial relationships that could be construed as a potential conflict of interest.
